# New Trends in Uniportal Video-Assisted Thoracoscopic Surgery for Primary Spontaneous Pneumothorax: A Narrative Review

**DOI:** 10.3390/jcm14061849

**Published:** 2025-03-09

**Authors:** Kenji Tsuboshima, Masatoshi Kurihara, Kota Ohashi

**Affiliations:** Pneumothorax Research Center and Division of Thoracic Surgery, Nissan Tamagawa Hospital, 4-8-1 Seta Setagaya-ku, Tokyo 158-0095, Japan; kuri@tf6.so-net.ne.jp (M.K.); m06014o@yahoo.co.jp (K.O.)

**Keywords:** video-assisted thoracoscopic surgery, uniportal, primary spontaneous pneumothorax, minimally invasive surgery, single-incision VATS, subxiphoid approach, intercostal approach

## Abstract

**Background:** Minimally invasive thoracic surgery has advanced since the introduction of multiportal video-assisted thoracoscopic surgery (mVATS) in 1991. Primary spontaneous pneumothorax (PSP) is an ideal condition for refining minimally invasive techniques owing to its straightforward procedures and predictable bullae distributions. **Methods:** Uniportal VATS (uVATS), which involves a single incision, is an alternative to mVATS, offering reduced postoperative pain, lower paresthesia rates, and comparable recurrence outcomes. This review explores two main uVATS approaches: intercostal and subxiphoid. **Results:** The intercostal approach is common to surgeons trained in mVATS, easier to adopt, and provides excellent cosmetic outcomes. Innovations such as the chest wall pulley method and anchoring sutures further enhance its operability and prevent recurrence. Subxiphoid uVATS minimizes intercostal nerve damage and postoperative pain, making it advantageous for bilateral PSP surgeries. However, it poses challenges such as longer operative times and limited dorsal visualization. Emerging strategies, including drainless postoperative management and two-lung ventilation with CO_2_ insufflation, have reduced surgical invasiveness. Additionally, cosmetic techniques such as subaxillary incisions enhance patient satisfaction. **Conclusions:** uVATS continues to redefine PSP surgery, prioritize patient-centered outcomes, and integrate novel strategies to achieve superior results.

## 1. Introduction

Spontaneous pneumothorax (SP) is a common condition encountered by thoracic surgeons, with primary spontaneous pneumothorax (PSP) being the most prevalent subtype. PSP typically occurs in young men without underlying lung disease. The main cause is the rupture of bullae formed at the lung apex or in segment 6 of the lung [1,2,3].

Conservative therapies, such as simple aspiration or chest tube drainage, are less invasive than surgery but have high recurrence rates, ranging from 29.2% to 57.9% [4,5]. Consequently, several guidelines recommend surgical treatment for patients with recurrent PSP [1,2,3].

Bullectomy via video-assisted thoracoscopic surgery (VATS) is the standard treatment for PSP, offering significantly lower recurrence rates (1.3% to 16.5%) than conservative methods [1,2,3,6,7]. Multiportal VATS (mVATS), primarily performed as a three-port VATS, remains the standard technique for SP, particularly PSP. The widespread adoption of this approach is attributed to the use of three ports, each serving a distinct purpose: one for the thoracoscope, one for grasping forceps to handle the lung, and one for autosutures to resect the bullae.

Recently, efforts to minimize surgical invasiveness have intensified, including thoracic surgery. PSP presents the following circumstances:

1. The surgical procedure is relatively straightforward.
2. The common sites of bullae responsible for PSP are well known, facilitating standardized surgical techniques.
3. Patients with PSP are often young and require a quick return to daily activities, such as education or work.

These factors have contributed to the emergence of uniportal VATS (uVATS) as a promising minimally invasive option for PSP surgery.

Initially, the primary advantage of uVATS was its single incision, and although the incision size in uVATS was double that in mVATS, it was acceptable. However, performing mVATS is the standard practice, and the need for additional value has led to a focus on reducing incision size, minimizing pain, improving operability, and preventing postoperative recurrence. Furthermore, new approaches aimed at alleviating patient burden have been explored, such as omitting intraoperative chest drain placement—previously considered standard—or injecting CO_2_ into the pleural cavity to reduce postoperative complications.

## 2. Conventional mVATS

The advent of the thoracoscope and autosuture in 1991 marked the development of mVATS technique in minimally invasive surgery [8,9]. Three-port VATS, the most commonly performed method, requires three skin incisions, each approximately 10–15 mm long. Compared with axillary thoracotomy, it offers superior cosmetic outcomes and reduces postoperative pain. However, its higher recurrence rates [10,11] have led to the adoption of various intraoperative measures, such as covering techniques, pleurodesis, and pleurectomy, to mitigate the issue [1,2,3,6,7,12,13,14,15]. Despite these challenges, the balance between cosmetic benefits and acceptable recurrence rates has established three-port VATS as the standard procedure for PSP [9].

As this technique has become more widely adopted, the demand for less invasive surgical approaches has increased. Recently, minimally invasive approaches other than three-port VATS have been reported. Initially, facilities adopted reduced-port and needlescopic surgeries to minimize the size of the ports [16,17]. Currently, uVATS has emerged as the leading minimally invasive thoracic surgery, offering fewer visible scars and reduced postoperative pain.

This transition to uVATS has been supported by advancements in surgical instruments such as thinner thoracoscopes, articulating graspers, and novel tools that replace traditional graspers. These innovations have helped establish uVATS as a mainstream technique for PSP treatment, fulfilling the evolving demand for minimally invasive surgery.

## 3. uVATS

This technique was first reported by Yamamoto et al. and Rocco et al. [18,19]. Typically, a thoracoscope, grasping forceps, and autosutures are simultaneously inserted through a single incision, approximately 20–30 mm long. Initially, the relatively larger incision was regarded as a limitation compared with the smaller incisions used in mVATS. Furthermore, surgical manipulation was challenging owing to the interference between the thoracoscope and other devices inside and outside the thoracic cavity. Over time, these obstacles have been addressed through advancements in technology and techniques.

uVATS can be broadly categorized into two main approaches: intercostal and subxiphoid, each offering distinct features and advantages. The approaches are described in the following sections.

### 3.1. Intercostal Approach

Similar to the conventional mVATS, this approach accesses the thoracic cavity via an intercostal incision in the lateral chest wall (Table 1). Leveraging thoracic surgeons’ prior experience is often the most straightforward method to adopt. Additionally, as the scar is invisible from the front, it is less conspicuous, potentially enhancing patient satisfaction.

In a 2015 review of eight studies comparing uVATS and mVATS in thoracic surgery, Akter et al. highlighted the advantages of uVATS for SP [20]. Among the four studies specifically addressing SP, this review revealed that along with its cosmetic benefits, uVATS was associated with reduced postoperative pain and a lower incidence of paresthesia. Similarly, Yang et al. conducted a comprehensive systematic review and meta-analysis of nine studies examining 768 patients with pneumothorax [21]. Their findings showed no significant differences in complication or recurrence rates between uVATS and three-port VATS. Moreover, Yang’s analysis and an independent study by Fiorelli et al. noted that uVATS contributed to reduced paresthesia rates and postoperative pain, particularly at 24 and 72 h after surgery [21,22]. The potential of uVATS in reducing early postoperative pain was further supported by Young et al., who reviewed 12 studies in 2015 [23]. This analysis, which included patients undergoing minor thoracic surgeries, such as SP, suggested that uVATS offers a slight advantage over mVATS in pain relief during the first 72 h post-surgery. However, the clinical significance of this benefit, typically reflected by a 1–2 point difference on the visual analog scale (VAS), remains limited. Nevertheless, these findings are consistent with those of other studies demonstrating the safety and efficacy of uVATS, further establishing it as a viable minimally invasive alternative to mVATS.

In a 2020 study, Kim KS evaluated the efficacy of a polyglycolic acid (PGA) sheet with fibrin glue in 59 patients undergoing uVATS for PSP [24]. A single 15–25 mm incision was made in the fourth or fifth intercostal space, and after wedge resection, the staple line was reinforced without pleural abrasion. The mean operation time was approximately 42 min, and the overall recurrence rate was 5.0%. No major perioperative complications were observed. The authors concluded that PGA sheet and fibrin glue reinforcement effectively prevented recurrence and minimized morbidity, highlighting uVATS as a safe and feasible option for PSP. Chuang et al. explored another aspect of uVATS by comparing its outcomes with those of needlescopic VATS in a cohort of 151 patients with PSP in 2024 [25]. Of these, 91 underwent uVATS through a single 25 mm incision along the fifth intercostal space, whereas 60 underwent needlescopic VATS. The uVATS group had significantly lower pain scores on the day of surgery (1.74 ± 1.35 vs. 2.65 ± 1.59). The recurrence rates were comparable between the groups (3.3% vs. 5.0%); however, the reduced pain in the uVATS group underscores its potential advantage. In a 2024 investigation, Janssen et al. compared 141 mVATS and 71 uVATS cases [26]. Their findings reaffirmed the advantages of uVATS, including shorter chest drain duration (median, 4 vs. 5 d) and hospital stay (median, 5 vs. 6 d). The recurrence rates were identical between the two groups (6%), and complication rates were comparable (uVATS: 11% vs. mVATS: 14%). uVATS facilitated earlier recovery and reduced healthcare costs while maintaining safety and efficacy comparable to mVATS. In 2024, Zhong et al. introduced an innovative variation in uVATS involving C-shaped cautery pleurodesis [27]. Compared with iodine-based chemical pleurodesis in patients with PSP, this technique demonstrated significantly lower postoperative VAS pain scores on days 1 and 2 and reduced postoperative drainage volumes. Both techniques exhibited low recurrence rates within 1 year (1.5% vs. 6.4%).

**Table 1:** Reviewed articles and summary of intercostal approach via uVATS for spontaneous pneumothorax.

Authors (Years)	Comparison (Cases)	Main Results	uVATS Details	Conclusions
Son et al. (2015) [28]	Anchoring suture technique (104)	Recurrence: 0.9%; mean operative time: 49.7 min; mean VAS score: 1.7 (post-op day 2)	Anchoring suture technique via <20 mm incision	Anchoring sutures improved operability and achieved low recurrence with minimal pain.
Han et al. (2016) [29]	TLVA with CO_2_ insufflation (130)	Recurrence: 3.8%; no severe adverse events; operative time: 30.9 min	25 mm incision, TLVA with SILS port, and CO_2_ insufflation	TLVA with CO_2_ improved visibility and reduced anesthesia-related risks.
Tsuboshima et al. (2015) [30]	uVATS with chest wall pulley method (23) vs. three-port VATS (102)	Shorter operative time: 71.7 min vs. 85.9 min; recurrence: 0% vs. 11.8% (insignificant)	20 mm incision, pulley method with ORC sheets, and fibrin glue for reinforcement	Chest wall pulley method improved operability and reduced operative time, maintaining comparable safety.
Lee et al. (2018) [31]	uVATS with CO_2_ (40) vs. without CO_2_ (40)	Better visibility and operative field in the CO_2_ group; operative time longer in the CO_2_ group	15–25 mm incision, CO_2_ insufflation	CO_2_ improved visibility but increased operative time and costs.
Kim et al. (2020) [24]	uVATS with PGA sheet + fibrin glue (59 cases; PSP)	Operation time: 41.6 ± 9.5 min; recurrence: 5.0%	15–25 mm incision, staple line reinforced with PGA sheet + fibrin glue	PGA sheet + fibrin glue effectively prevents recurrence without pleural abrasion.
Fiorelli et al. (2021) [22]	uVATS with additional puncture (21) vs. three-port VATS (22)	Lower VAS scores and paresthesia rates in the uVATS group; high patient satisfaction	20–25 mm incision with additional anchoring suture	uVATS improved pain and satisfaction while maintaining safety.
Lee et al. (2022) [32]	uVATS with spinal needle anchoring (139)	Recurrence: 2.2%; operative time: 36.7 min; VAS score: low	15–20 mm incision, spinal needle anchoring technique	Spinal needle anchoring enhanced operability and reduced pain and recurrence.
Chuang et al. (2024) [25]	uVATS (91) vs. needlescopic VATS (60)	Lower pain scores on surgery day: 1.74 vs. 2.65 d; recurrence: 3.3% vs. 5.0%	25 mm incision, mechanical pleurodesis	uVATS demonstrated better pain relief on the day of surgery with similar recurrence rates.
Janssen et al. (2024) [26]	uVATS (71) vs. mVATS (141)	Recurrence: 6% both groups; complication rates: 11% vs. 14%; shorter hospital stay: 5 vs. 6 d	30–40 mm incision for bullectomy	uVATS was equally safe and effective, with shorter recovery times.
Takamori et al. (2024) [33]	Drainless uVATS (54)	Recurrence: 3.7%; median hospital stay: 1 d; no re-interventions	18–20 mm incision, drainless management	Drainless uVATS promoted early discharge and demonstrated safety.
Zhong et al. (2024) [27]	uVATS with C-shaped cautery pleurodesis (65) vs. chemical pleurodesis (63)	VAS scores and drainage volumes lower in the C-shaped group; recurrence: 1.5% vs. 6.4%	30 mm incision with C-shaped electrocautery rings	C-shaped method reduced postoperative pain and drainage, showing potential benefits.

Abbreviations: ORC, oxidized regenerated cellulose; PGA, polyglycolic acid; SILS, single-incision laparoscopic surgery; TLVA, two-lung ventilation anesthesia; VAS, visual analog scale; min, minutes; d, days.

#### 3.1.1. Improving Operability

Device collisions and limited surgical visualization often pose challenges in uVATS compared with mVATS. To address these issues, Tsuboshima et al. introduced a novel technique in 2015 that utilizes threads instead of graspers to minimize device interference and reduce incision size [30,34]. This initial method incorporated a chest wall pulley, which allowed precise manipulation of lesions in any direction, resembling a marionette. In 2017, the technique was refined by introducing a dual pulley, which further enhanced its precision and operability. In their study, 23 patients underwent uVATS using the pulley method, whereas 102 underwent three-port VATS, with propensity score matching applied for comparison. In the uVATS group, a 20 mm incision was made in the fifth intercostal space along the anterior axillary line. Two chest wall pulleys controlled the traction threads, enabling precise lesion positioning while avoiding instrument collision. The lesions were resected using endoscopic staplers, and oxidized regenerated cellulose sheets and fibrin glue were applied to reinforce the pleura and prevent recurrence. The uVATS group exhibited shorter operative times (71.7 ± 15.7 min vs. 85.9 ± 25.5 min) and required fewer staplers (3.6 ± 1.2 vs. 4.5 ± 1.2). The recurrence rates were 0% (0/17) and 11.8% (2/17) in the uVATS and three-port VATS groups, respectively; however, the difference was insignificant. In 2015, Son et al. proposed a simplified technique that utilized threads anchored to the lung to pull lesions toward the port in a single direction [28]. This approach involved making an incision of less than 20 mm at the fourth or fifth intercostal space along the anterior axillary line. After the apical bullae were identified, anchoring sutures were placed anterior to the lesions, allowing the lung to be pulled and stabilized for wedge resection using staplers. The staple line was reinforced with PGA sheets and fibrin glue. In their study of 104 PSP cases, the procedure showed excellent outcomes, with an average operative time of 49.7 ± 13.9 min, a postoperative length of hospital stay of 4.8 ± 1.7 d, and a recurrence rate of 0.9%. Postoperative pain, measured using VAS scores, was well controlled and remained at a mean value of 1.7 on the second day.

Some broader interpretations of this technique (not strictly uVATS) involve additional punctures to enhance operability. For example, in 2021, Fiorelli et al. reported a modified uVATS technique that added a single puncture to the traction sutures to improve surgical handling [22]. They compared 21 patients who underwent the modified uVATS approach with 22 patients who underwent three-port VATS. A 20–25 mm skin incision was made at the fifth intercostal space along the anterior axillary line, and a traction suture was inserted through another small puncture at the third intercostal space along the anterior axillary line. The suture was passed through the lung parenchyma near the lesion and pulled out through the main incision. The modified uVATS group showed significantly lower VAS pain scores at 24, 48, and 72 h postoperatively. Additionally, the incidence of postoperative paresthesia was significantly lower, and patient satisfaction scores were higher in the uVATS group. In 2022, Lee et al. introduced an innovative technique using a spinal needle to enhance the safety and operability of PSP surgery [32]. This method involved creating a 15–20 mm skin incision at the fifth intercostal space along the mid-axillary line, through which a 5 mm 30° thoracoscope and staplers were inserted. A 20 G, 90 mm spinal needle was then introduced through a separate puncture at the second intercostal space along the anterior axillary line. The needle was used to hook and stabilize the bullae, thereby facilitating wedge resection using staplers. The staple line was reinforced with PGA sheets and fibrin glue. Among the 139 PSP cases studied, the mean operative time was 36.69 ± 14.64 min (median: 30 min), with a postoperative chest drain duration of 1.97 ± 0.77 d and a hospital stay of 3.00 ± 0.78 d. The recurrence rate was 2.2% (3/139), and no postoperative complications were observed.

#### 3.1.2. Further Minimally Invasive Approaches with Alternative Strategies

Many intercostal approaches for uVATS emphasize reducing the incision size; however, alternative strategies have been developed to further minimize invasiveness.

##### Drainless Postoperative Management

Takamori et al. reported a drainless uVATS approach in 2024 aiming to further reduce surgical invasiveness [33]. This retrospective study included 54 patients with PSP from two facilities. A single incision measuring 18–20 mm was made in the fourth or fifth intercostal space along the anterior axillary line. The procedure utilized a 5 mm 30° rigid thoracoscope, articulating graspers, and autosutures to resect the apical bullae. The staple line was reinforced with soft coagulation or fibrin glue as necessary. An air leak test was performed at 15–20 cmH_2_O, and any detected leaks were addressed using additional suturing, coagulation, or glue application. If no air leaks were observed, the procedure was completed without the placement of a chest drain. None of the patients required reoperation or redrainage. The median operative time was 42 min, with a median hospital stay of 1 d. The recurrence and complication rates were both 3.7%, demonstrating the feasibility and safety of this approach.

##### Two-Lung Ventilation Anesthesia (TLVA) with CO_2_ Insufflation

Traditionally, mVATS under one-lung ventilation anesthesia has been the standard approach for PSP. However, uVATS under TLVA combined with CO_2_ insufflation has emerged as a less invasive alternative, potentially reducing the risks associated with general anesthesia.

In 2016, Han et al. evaluated the safety and efficacy of uVATS using TLVA and CO_2_ insufflation in 130 patients with PSP [29]. A single 25 mm incision was made in the sixth or seventh intercostal space, through which a single-incision laparoscopic surgery (SILS) port was inserted. Continuous CO_2_ insufflation at 6 mmHg was maintained to ensure a clear surgical field. The procedure utilized a 5 mm thoracoscope, flexible endoscopic staplers, and articulating forceps, with all patients undergoing mechanical and chemical pleurodesis. The mean operative time was 30.9 ± 8.2 min, and no severe complications related to CO_2_ insufflation or TLVA were observed. The recurrence rate was 3.8%. This study emphasized that CO_2_ insufflation enhances visualization, enabling shorter operative and anesthesia durations. Furthermore, the avoidance of a double-lumen tube, which is required for one-lung ventilation anesthesia, reduces risks such as hypoxemia and intubation-related complications. In 2018, Lee et al. further assessed the safety and outcomes of uVATS with CO_2_ insufflation in a cohort of patients aged 19–64 years with SP, including those with PSP [31]. Participants were divided into two groups: a CO_2_ insufflation group (Group C, 40 patients) and a non-CO_2_ insufflation group (Group NC, 40 patients). A single 15–25 mm incision was made in the sixth or seventh intercostal space. In Group C, CO_2_ insufflation at 6 mmHg was delivered via a SILS port. The bullae were resected using endoscopic instruments and a 5 mm thoracoscope with a staple line reinforced using PGA sheets and fibrin glue. CO_2_ insufflation improved visualization during thoracoscope insertion, particularly at the beginning of the procedure. However, longer operative times and mild increases in PaCO_2_ and reductions in oxygenation were observed, although these changes remained within safe limits for young, healthy patients. The use of specialized ports and instruments (such as SILS ports) increases costs, posing a challenge for widespread adoption.

#### 3.1.3. Optimizing Cosmetic Outcomes

In 2023, Zheng et al. compared the subaxillary cosmetic incision (SACI) approach in 21 patients with PSP and the intercostal approach for uVATS in 57 patients [35]. After propensity score matching, 21 patients were analyzed in each group. The SACI technique involves a 25 mm incision placed discreetly in the axillary fold at the third intercostal space. A 5 mm 30° thoracoscope was used to locate the bullae, which were resected using an endoscopic stapler. In the uVATS group, a 25 mm incision was made in the fourth or fifth intercostal space, extending from the anterior to the mid-axillary line, with the same procedural steps applied. Postoperative pain scores, hospital stay duration, and recurrence rates (with no recurrence observed in either group during the 6-month follow-up period) were comparable between the two groups. However, SACI offers a distinct advantage in that the incision situated in the axillary region remains concealed when the arm is lowered. This feature contributed to significantly higher patient satisfaction with scarring at both 2 weeks and 6 months postoperatively.

### 3.2. Subxiphoid Approach

The subxiphoid approach is commonly used for anterior mediastinal tumors; however, it is relatively rare in PSP treatment (Table 2). Unlike the intercostal approach, this approach is less likely to cause intercostal nerve damage, making it associated with minimal postoperative pain. Additionally, for simultaneous bilateral surgeries, the subxiphoid approach allows the procedures to be performed using the same incision, which is a distinct advantage [36,37]. However, as the incision is located on the anterior side of the patient, it may be cosmetically less favorable [37]. Furthermore, this approach offers a surgical view that is entirely different from intercostal approaches such as those used in mVATS. This difference requires familiarity with the technique and raises concerns regarding the limited visualization of the dorsal regions, particularly segment 6, which is a common bullae site.

In 2016, Li et al. conducted a randomized controlled trial comparing 22 cases of subxiphoid uVATS with 21 cases of intercostal uVATS for the SP [37]. The subxiphoid approach involved a 30–40 mm vertical incision below the xiphoid process, whereas the intercostal approach utilized a 30–40 mm incision at the fourth or fifth intercostal space. Patients who underwent subxiphoid uVATS experienced significantly less postoperative pain on days 0, 1, 2, and 3 than those who underwent intercostal uVATS. However, the subxiphoid approach required significantly longer operative times (52.50 ± 14.55 min vs. 34.29 ± 11.53 min). Recurrence was observed in one case in the subxiphoid group and in none of the cases in the intercostal group. In 2016, Wang et al. evaluated whether subxiphoid uVATS could significantly reduce postoperative pain compared with intercostal uVATS (26 cases) and three-port VATS (17 cases) for PSP [38]. In the subxiphoid uVATS group, a 20 mm vertical incision was made below the xiphoid process. Endoscopic staplers were used for the bullectomy, followed by mechanical pleurodesis. For intercostal uVATS, a 20 mm incision was made in the fifth intercostal space along the anterior axillary line. In the three-port VATS group, a 10 mm video port was placed at the seventh intercostal space, and two 5 mm ports were positioned at the anterior axillary line of the fifth intercostal space and the posterior axillary line of the sixth intercostal space. The subxiphoid uVATS group had significantly lower postoperative pain scores at 1 h and 8 h than the other two groups. The operative time was longer in the subxiphoid uVATS group; however, no significant differences were observed among the three groups in postoperative complications or length of hospital stay. The recurrence rates were low across all procedures: 7.1% for subxiphoid uVATS, 11.5% for intercostal uVATS, and 11.8% for three-port VATS. In 2019, Chen et al. retrospectively compared 32 uVATS cases using the subxiphoid approach with 95 three-port VATS cases [39]. The subxiphoid approach involved a skin incision of approximately 30 mm. After propensity score matching, the 32 patients in each group were compared. The recurrence rate was 3.1% in both groups. However, the operative time was significantly longer in the subxiphoid group (80.47 ± 27.04 min vs. 57.31 ± 34.95 min), and the incidence of intraoperative arrhythmias was significantly higher in the subxiphoid group (21.9% vs. 0.0%).

## 4. Limitations

This review has several limitations. First, the studies we summarized vary in design (retrospective analyses, small-scale randomized trials) and patient populations, making direct comparisons challenging. Many of the cited studies include relatively small sample sizes and short follow-up periods, limiting the generalizability of their findings. Second, we did not perform a formal risk-of-bias assessment or employ structured tools such as the GRADE framework or the Newcastle–Ottawa Scale to evaluate the quality of evidence. As a result, it is difficult to draw definitive conclusions about the strength of each study’s findings. Third, while most studies reported postoperative pain and recurrence rates, other important outcomes such as quality of life, long-term functional status, and comprehensive cost-effectiveness were not consistently addressed. This inconsistency highlights the need for larger, more methodologically rigorous investigations to unify outcome reporting. Future research should also standardize assessment tools and follow-up protocols to facilitate more robust comparisons across different surgical approaches for primary spontaneous pneumothorax.

## 5. Conclusions

The transition toward minimally invasive surgery for PSP has accelerated in recent years, with uVATS being established as the mainstream approach. This technique provides considerable cosmetic advantages while reducing postoperative pain and paresthesia, and numerous studies have confirmed its comparable efficacy to mVATS in recurrence prevention. However, the focus has evolved beyond the achievements of single-incision techniques. Reducing the incision size remains a logical and achievable goal, whereas innovative approaches, such as drainless postoperative management and two-lung ventilation with CO_2_ insufflation, have introduced new opportunities to further minimize surgical invasiveness.

## 6. Future Directions

As minimally invasive approaches continue to evolve, several promising areas warrant further exploration:

1. Refinement of uVATS Techniques: Future studies should focus on refining existing innovations, such as the chest wall pulley method, to improve operability and efficiency while maintaining safety and efficacy.
2. Optimizing Patient Outcomes: Research on drainless postoperative management and TLVA with CO_2_ insufflation should prioritize the reduction of postoperative pain, length of hospital stays, and complications, particularly in younger patients. Additionally, incorporating proper physical activity protocols could support mental health and accelerate return to daily activities, especially for adolescents and young adults with PSP [40].
3. Cosmetic Advancements: Techniques such as subaxillary incisions have shown promise in improving patient satisfaction. Continued innovations in incision placement and scar concealment could further enhance cosmetic outcomes.
4. Long-Term Outcomes: Robust large-scale studies assessing the long-term safety, recurrence rates, and cost-effectiveness of uVATS compared with mVATS and other techniques are essential to guide clinical practice.

## Figures and Tables

**Table 2:** Reviewed articles and summary of subxiphoid approach using uVATS for spontaneous pneumothorax.

Authors (Years)	Comparison (Cases)	Main Results	uVATS Details	Conclusions
Li et al. (2016) [37]	Subxiphoid uVATS (22) vs. intercostal uVATS (21)	Pain was lower in the subxiphoid group (days 0–3); operative time: 52.50 ± 14.55 min vs. 34.29 ± 11.53 min; recurrence: 4.5% vs. 0%	30–40 mm subxiphoid vertical incision	Subxiphoid approach reduced early postoperative pain but required longer operative time.
Wang et al. (2016) [38]	Subxiphoid uVATS (14) vs. intercostal uVATS (26) vs. three-port VATS (17)	Pain was lower in the subxiphoid group at 1 and 8 h post-surgery; recurrence: 7.1% vs. 11.5% vs. 11.8%	20 mm subxiphoid vertical incision, mechanical pleurodesis	Subxiphoid uVATS effectively reduces early postoperative pain with comparable recurrence rates.
Chen et al. (2019) [39]	Subxiphoid uVATS (32) vs. three-port VATS (95)	Recurrence: 3.1% in both groups; operative time: 80.47 ± 27.04 min vs. 57.31 ± 34.95 min; arrhythmias: 21.9% vs. 0.0%	30 mm subxiphoid incision	Subxiphoid approach had similar recurrence rates to three-port VATS but longer operative time and higher arrhythmia rates.

Abbreviations: uVATS, uniportal video-assisted thoracoscopic surgery; min, minutes; d, days.

## Data Availability

No new data were created or analyzed in this study.

## Abbreviations

The following abbreviations are used in this manuscript:

mVATS	Multiportal video-assisted thoracoscopic surgery
PGA	Polyglycolic acid
PSP	Primary spontaneous pneumothorax
SACI	Subaxillary cosmetic incision
SILS	Single-incision laparoscopic surgery
SP	Spontaneous pneumothorax
TLVA	Two-lung ventilation anesthesia
uVATS	Uniportal video-assisted thoracoscopic surgery
VAS	Visual analog scale
